# Time After Time: A Second Look at Evolving Rash With Multiple Exposures to Amoxicillin

**DOI:** 10.7759/cureus.17807

**Published:** 2021-09-07

**Authors:** Ozioma Nnomadim, Heather Speck, Daniela Vidaurri

**Affiliations:** 1 Medicine, University of the Incarnate Word School of Osteopathic Medicine, San Antonio, USA; 2 Pediatrics, Baylor College of Medicine, San Antonio, USA

**Keywords:** exanthematous drug eruption, serum sickness-like reaction, erythema multiforme, urticaria multiforme, urticarial vasculitis, acute hemorrhagic edema of infancy, henoch-shonlein purpura.

## Abstract

Pediatric penicillin drug reactions may present in many forms such as erythema multiforme, serum-sickness, serum-sickness-like reaction (SSLR), Henoch-Schonlein Purpura (HSP), and urticarial vasculitis. Here, we review the case of a 13-month-old with atypical presentation of a drug reaction with increasing severity after each exposure to amoxicillin. We discuss the various differential diagnoses in comparison to our patient’s presentation and conclude with the recommendation of considering timing and previous exposures in the diagnosis of drug-associated rashes in pediatric population.

## Introduction

The etiologies of childhood exanthems range from infectious processes to non-infectious processes such as vasculitides and drug-induced allergic reactions. Penicillin use for the treatment of acute otitis media in the pediatric population is very common due to its efficacy in comparison to other medical therapies [1], however, penicillin resistance and hypersensitivity are also becoming very common [2]. Penicillin-induced drug reaction in the pediatric population varies in severity and can be very unnerving to parents and caregivers. One of the presenting signs of penicillin-drug reaction is drug rash in the forms of erythema multiforme, serum-sickness reaction, serum-sickness-like reaction (SSLR), Henoch-Schonlein Purpura (HSP), urticarial vasculitis, and morbilliform eruption. Here, we review the case of a 13-month-old with increasing severity of drug reaction with each exposure to amoxicillin. We compare the natural progression of her rash to different possible cutaneous drug reactions and consider associated laboratory findings.

## Case presentation

The patient is a full term, previously healthy 13-month-old infant female who presented to the emergency department (ED) with diffuse urticarial purpuric rash. According to her father, the patient had a viral upper respiratory infection (URI) that was complicated by acute otitis media which necessitated treatment with amoxicillin. The patient was started on a 10-day course of amoxicillin but developed a mild urticarial rash on the trunk and diaper region on the seventh day. The patient had a similar experience with initial use of amoxicillin in the first year of life but with milder presentation and spontaneous resolution within 24 h. Amoxicillin was immediately discontinued. On day 8, the rash changed in morphology to palpable purpura and progressed to the head, neck, and all extremities while sparing the palms, soles, and mucosal surfaces. The patient was brought to the ED and admitted to past medical history (PHM) with a tentative diagnosis of Erythema multiforme. By day 9, the original purpuric lesions began to fade to ecchymosis, while a new wave of intensely pruritic urticaria erupted. The new lesions were blanchable and lacked purpura like originally seen. Complete blood count and C-reactive protein (CRP) were both unremarkable. The patient was started on prednisone and diphenhydramine hydrochloride (HCL), however, rash continued to progress with poor control of pruritus. The patient eventually had symptomatic relief with hydroxyzine while the second dose of prednisone stopped the eruption of new rashes.

Physical examination findings

**Figure 1:**
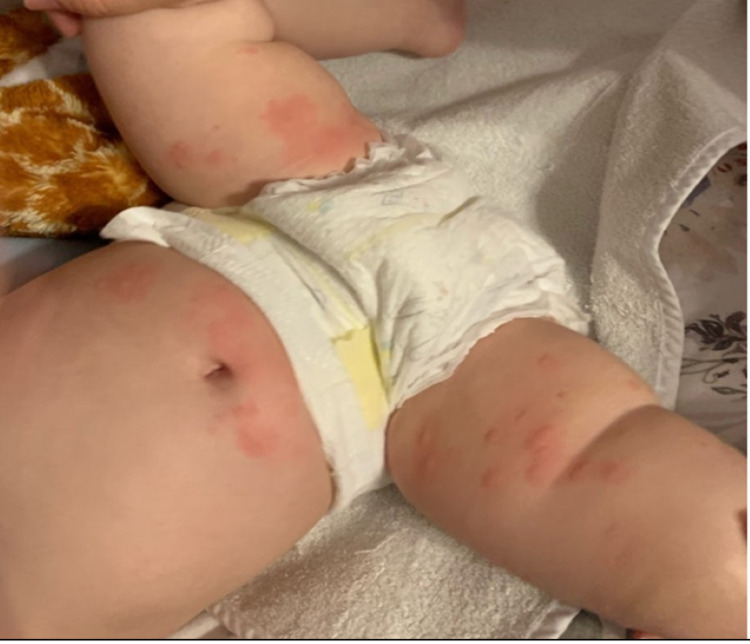
Rash day 1 (morning). Day 7 of amoxicillin use. Patient presents with a polycyclic blanchable erythematous rash mostly on the trunk and lower extremities.

**Figure 2:**
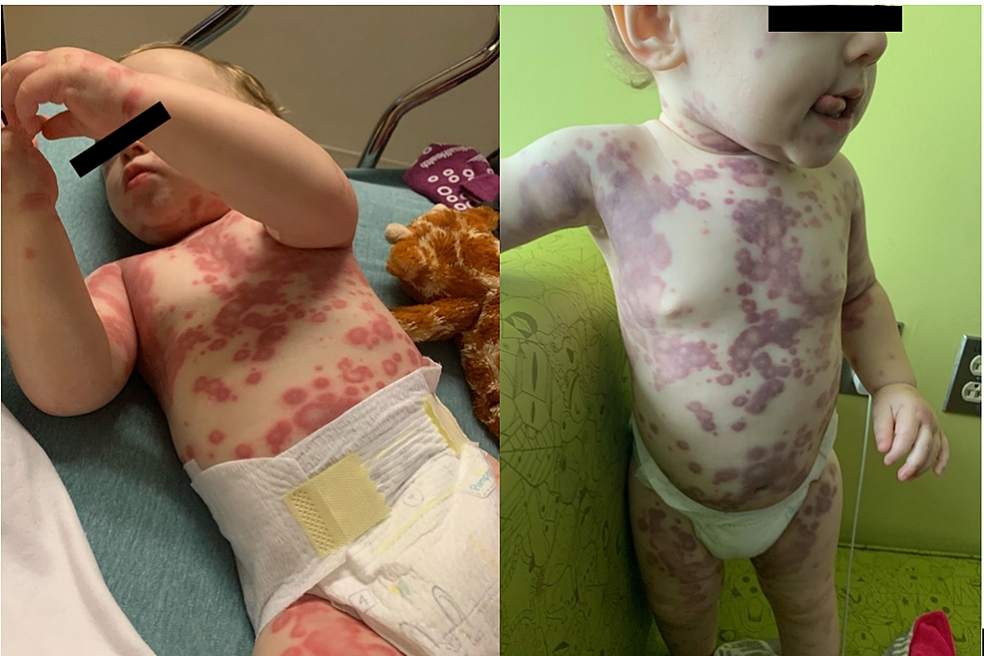
Left - rash day 1 (evening), day 7 of amoxicillin use. Right - rash day 2 (morning), amoxicillin discontinued. Left: palpable purpura noted on admission to hospital. Right: original purpura fading into ecchymosis.

**Figure 3:**
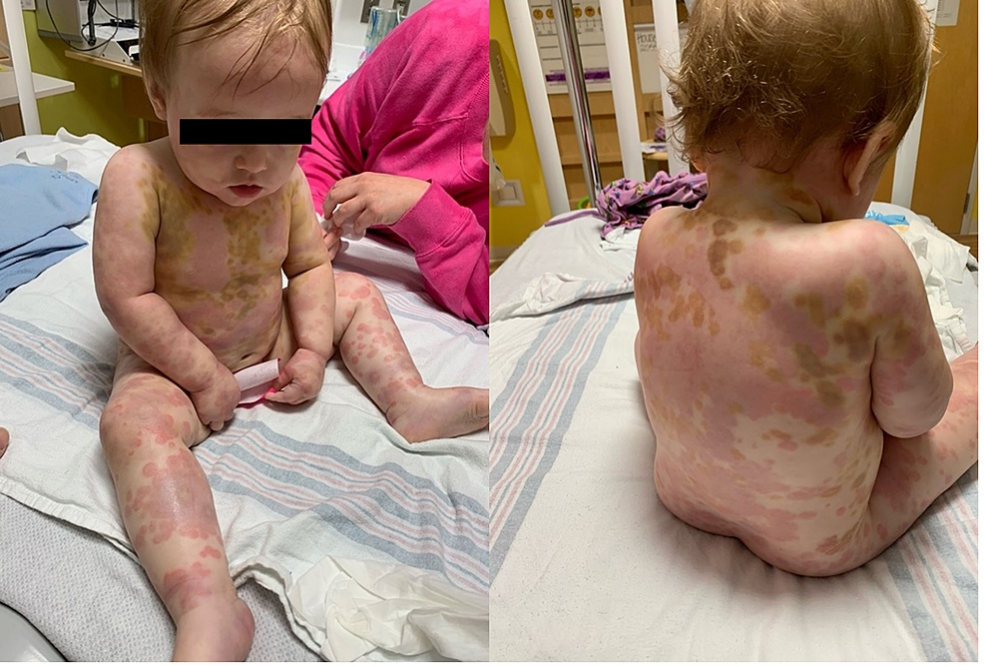
Rash day 3, prednisone initiated. Left: Fading of purpura to ecchymosis; Right: New eruption of pruritic blanchable wheals.

Figure 1 shows initial presentation of rash. Figure 2 shows evolving rash after amoxicillin was discontinued and treatment with prednisone was started. Figure 3 shows rash before the administration of second dose of prednisone.

## Discussion

Given the varied morphologic features of our patient’s rash and unique timeline of progression, several systemic and dermatologic conditions were taken into consideration when forming our differential diagnosis including exanthematous drug eruption, SSLR, erythema multiforme, urticaria multiforme, urticarial vasculitis, acute hemorrhagic edema of infancy, and Henoch-Shonlein purpura (HSP). We will discuss each diagnosis separately and compare to our patient’s presentation. 

Exanthematous drug eruption is the most common type of cutaneous drug eruption in children, and typically presents in a morbilliform distribution consisting of fine maculopapular lesions that originate centripetally and travel to the extremities [3]. This form of drug reaction will typically occur one to two weeks after initiation of the offending drug, with antibiotics, nonsteroidal anti-inflammatory drugs (NSAIDs), barbiturates, phenytoin, carbamazepine, and benzodiazepines being the most common culprits [3]. Much like our patient presented, these eruptions can be exacerbated by viral illness, and may also present with pruritus and mild fevers. While our patient shared similar historical characteristics with this type of drug eruption, her rash was not macular or papular in morphology [4]. SSLR is a hypersensitivity reaction to an offending drug which typically has a delayed onset following antigen exposure. Symptoms typically include rash, fever, myalgias, lymphadenopathy, and arthritis, but differs from true serum sickness by lacking detectable circulating immune complexes or hypocomplementemia [5]. Laboratory findings include leukocytosis, elevated erythrocyte sedimentation rate (ESR), elevated CRP, and proteinuria. Cutaneous manifestations include urticaria and in rare cases may appear as a morbilliform or scalariform rash. Our patient’s presentation shares a lot of similarity and also lacks remarkable laboratory findings (aside from leukocytosis); skin biopsy provides definitive diagnosis.

Urticaria multiforme, also known as “Acute Annular Erythema,” is a cutaneous hypersensitivity reaction that is often preceded by viral infection and presents with polycyclic or annular urticarial plaques which then progress to bruising as rash subsides [3]. It is often misdiagnosed as erythema multiforme, but differs in the lack of bullae formation, skin necrosis, and mucosal membrane involvement. The diagnostic criteria include: annular to polycyclic urticarial lesions with transient ecchymotic skin changes, duration of individual lesions lasting less than 24 h, angioedema or acral edema, dermatographism, modest elevation of acute phase reactants, and a favorable response to antihistamines [6]. In comparison to urticaria multiforme, our patient only had the urticarial lesions with ecchymosis, but did not have the same duration of lesions, angioedema, acral edema, dermatographism, elevation in acute phase reactants, or favorable response to antihistamines until diphenhydramine was switched to hydroxyzine. Furthermore, our patient did not have the defining characteristics listed above for the diagnosis of erythema multiforme, although this was the initial diagnosis considered by the ER physician who decided on admission.

 Urticarial vasculitis is a type III hypersensitivity reaction mediated by antigen-antibody complexes deposited on vascular endothelium resulting in inflammation and vasculitis, and oftentimes precipitated by infections and medications [3]. Cutaneous manifestations are typically non-pruritic wheals with petechiae, purpura, and post-inflammatory hyperpigmentation. Symptoms usually present with fevers and arthralgias, and can even manifest with nephritis, uveitis, angioedema, and chest pain [7]. This can be distinguished from the previously discussed rashes by lesions that are fixed for more than 24 h, and that are described as burning or stinging, as opposed to pruritic. Not only is this rare in children, but our patient lacked the systemic vasculitides described and also only experienced pruritus with the second eruption of her rash.

While mentioning small vessel vasculitides, acute hemorrhagic edema of infancy (AHEI) was an additional possibility on the list of differential diagnoses. AHEI, also known as Finkelstein's or Seidlamayer's disease, is a cutaneous small vessel vasculitis of young children characterized by low grade fever, erythematous edema, and purpuric lesions mainly on the face and extremities [3]. Symptoms usually occur within two weeks of vaccine administration of tetanus, diphtheria, and pertussis (Tdap), measles, mumps and rubella (MMR), varicella, and Hib [8]. This is distinguished from our patient based on rash distribution and preceding events leading to eruption.

Lastly, we will discuss HSP, the most common multi-system vasculitis in children. HSP is also a small vessel vasculitis seen in small children following a viral illness and is thought by some to be a more severe relative to AHEI [3]. Cutaneous manifestations of HSP are palpable, non-thrombocytopenic purpura primarily seen on the lower extremities and buttocks with extracutaneous manifestations such as arthritis, nephritis, and gastrointestinal (GI) involvement [8]. Once again, our patient lacked systemic involvement that would be seen in HSP.

## Conclusions

Diagnosis of drug rash in pediatric populations remains very challenging due to multiple presentations with shared similarities and all dependent of the timing. The evolution of a rash was clearly very important in diagnosing this particular patient, who most likely had serum sickness-like reaction (SSLR) due to exposure to amoxicillin. Inquiring about prior descriptions of a rash or having pictures, can facilitate the diagnosis. Skin biopsy provides a definitive diagnosis but was not performed in this patient due to rash resolution following prednisone administration. This patient was initially thought to have erythema multiforme. Once admitted, her initial working diagnosis was HSP; and it was not until her second wave of hives appeared that her clinical picture became quite consistent with SSLR.
